# The Effect of Autism Spectrum Disorder on Family Mental Health: Challenges, Emotional Impact, and Coping Strategies

**DOI:** 10.3390/brainsci14111116

**Published:** 2024-11-01

**Authors:** José Jesús Sánchez Amate, Antonio Luque de la Rosa

**Affiliations:** Department of Education, University of Almeria, 04120 Almería, Spain; jsa819@ual.es

**Keywords:** Autism Spectrum Disorder (ASD), family and caregivers, mental and emotional health, coping strategies

## Abstract

Background: Autism Spectrum Disorder (ASD) impacts not only diagnosed individuals, but also significantly affects the quality of life of both primary and secondary caregivers. These effects are particularly pronounced when compared to caregivers of individuals with other neurodevelopmental disorders. The emotional and physical demands of caring for someone with ASD can profoundly alter family dynamics and interpersonal relationships, creating challenges that require a comprehensive approach to be understood and addressed. Methods: The methodological design is a narrative review study, based on a search conducted during May, June, July, and August 2024 in the Scopus, Dialnet, and WoS databases concerning the object of study. As a result, a total of 197 articles were qualitatively analyzed. Of these, 36 articles were selected for a more detailed qualitative analysis, leading to a final sample of 14 documents. The selected studies were examined through qualitative content analysis. The inclusion criteria for this selection were as follows: empirical studies or research published in English or Spanish; open access via the Internet; categories limited to “education/educational research” relevant to the proposed objectives; and specific documents related to students with Autism Spectrum Disorder (ASD). Results: The care of individuals with Autism Spectrum Disorder (ASD) has a significant and multifaceted impact on family life, deeply affecting the mental health of caregivers. These effects manifest in the form of chronic stress, anxiety, and interpersonal difficulties, altering family dynamics. The quality of life of caregivers varies depending on the coping strategies they employ, which are crucial for their emotional well-being. Conclusions: Understanding and optimizing these strategies is essential to mitigate the negative effects of caregiving and improving the overall well-being of families living with ASD.

## 1. Introduction

Autism Spectrum Disorder (ASD) is a multifaceted neurobiological condition that presents a variety of symptoms that profoundly affect communication, social interaction, and behavior [1]. While attention is often focused on the diagnosed individual, it is crucial to recognize that the implications of this condition extend considerably to the family unit [2]. Emotional well-being and family dynamics can undergo profound changes, creating unique challenges that require specific attention and support [3].

The diagnosis of a family member with ASD initiates a complex and often overwhelming adaptation process. Families must cope with understanding the diagnosis, seeking appropriate treatments, and adjusting to the specific needs of the individual with ASD [4]. This process can create a substantial burden, with significant implications for the mental health of all family members [5]. The stress resulting from continuous caregiving, the need to coordinate multiple specialist appointments, and the adaptation of the home environment can be overwhelming, affecting not only the parents but also other family members, who may feel that their own needs and experiences are being neglected [6].

The emotional impact of caring for an individual with ASD varies widely, often manifesting as anxiety, sadness, or guilt [7]. Parents may go through a grieving process related to unmet expectations and experience a sense of isolation due to a lack of understanding or support from their social environment [8]. The constant pressure to manage challenging behaviors associated with ASD can lead to fatigue and burnout, thereby deteriorating the mental health of primary caregivers [9]. Additionally, prolonged stress and a sense of lack of control can predispose parents to disorders such as anxiety and depression, affecting their ability to provide adequate emotional support to the individual with ASD [10].

The situation also affects other family members in complex ways. The attention and time that parents must devote to the individual with ASD can lead to conflicting feelings and confusion among other household members [11]. The perception that time and resources are disproportionately directed toward the individual with ASD may cause feelings of neglect among others, exacerbating their frustration and sadness [12]. These emotions can trigger significant emotional difficulties, such as anxiety and resentment, which in turn negatively affect their social and emotional development [13]. The constant imbalance may lead to communication problems and increase tension within the household, creating a stressful environment [14]. Neglecting the emotional needs of other family members can contribute to a cycle of prolonged stress, where each individual struggles to find their own balance while managing the impact of ASD on family dynamics [15]. This situation may lead to greater family dysfunction, affecting overall cohesion and the general well-being of all family members [16].

Despite these challenges, there are strategies and resources that can be highly beneficial in helping families manage the impact of ASD and improve their emotional well-being. Psychological intervention, which may include both individual and family therapy, has proven to be effective [17]. Family therapy has the potential to address altered dynamics and provide tools for effective communication and stress management [18]. On the other hand, individual therapy for parents can be crucial in addressing stress and the emotions related to caregiving for the individual with ASD [19]. Support groups for families offer a platform to share experiences, receive guidance, and find comfort in mutual understanding [20]. These groups can be an invaluable source of emotional and practical support, helping parents feel less isolated and more connected to others in similar circumstances [21].

In addition, providing families with education about ASD and effective strategies for behavior management is crucial for empowering them and reducing uncertainty [22]. Training in coping skills and stress management techniques can help families better handle daily demands and foster a more positive outlook [23]. Access to community resources, such as respite services, can offer temporary relief to caregivers, allowing them time for self-care, which is essential for maintaining a healthy balance between caring for the individual with ASD and the well-being of other family members [24].

To effectively address the impact of ASD on family mental health, it is essential to adopt a comprehensive approach that considers both the needs of the affected individual and those of their family [25]. Collaboration among healthcare professionals, educators, and the community can contribute to creating a supportive environment that benefits all family members [26]. Promoting a broader understanding of ASD and eliminating associated stigma are equally crucial [27]. Encouraging greater awareness and empathy in society can reduce isolation and pressure on families, facilitating a more inclusive and supportive environment [28].

In summary, the impact of Autism Spectrum Disorder (ASD) on family mental health is profound and multifaceted. The emotional and psychological challenges faced by family members require careful attention and appropriate support strategies [29]. Addressing these needs not only improves the quality of life for the individual with ASD, but also enhances the well-being and resilience of their family members [30]. Collaboration and access to adequate resources are crucial for establishing a supportive environment that promotes mental health and overall well-being for all family members [24].

In relation to the above, this research is based on and aims to understand the impact of Autism Spectrum Disorder (ASD) on family mental health, considering the complexity of the emotional and psychological challenges faced by families of individuals with ASD. This research seeks to analyze the emotional impact associated with raising and caring for a family member with ASD, as well as to explore the coping strategies that families employ to manage these challenges. Thus, the research questions posed to be addressed through this narrative review are the following:

- How does Autism Spectrum Disorder (ASD) affect the overall quality of life of primary and secondary caregivers compared to caregivers of children with other neurodevelopmental disorders?
- What effects does caring for a child with Autism Spectrum Disorder have on family dynamics and interpersonal relationships?
- What factors contribute to the variability in caregivers’ quality of life, and how do different coping strategies affect family members or caregivers of individuals with Autism Spectrum Disorder?

## 2. Materials and Methods

In accordance with the established objectives, a narrative review was conducted. This review aimed to evaluate the quality of life of primary and secondary caregivers of children with Autism Spectrum Disorder (ASD) compared to caregivers of children with other neurodevelopmental disorders. It also identified significant differences in their overall well-being. Additionally, the impact of caring for a child with ASD on family dynamics and interpersonal relationships was analyzed. Finally, the factors influencing the variability in caregivers’ quality of life were explored, and the different coping strategies affecting their well-being and that of their families were assessed. The Scopus, Dialnet, and WoS (Web of Science) databases were utilized for this purpose. This methodology aims to provide a comprehensive and detailed analysis of the available information on the subject. The selection of these databases was justified by their international prestige, academic recognition, and focus on educational research [31].

The process began with an exhaustive search in these databases, followed by the collection and selection of relevant articles. After this initial stage, a detailed analysis was performed, extracting key descriptive information from each study, including author, objective, methodology, participants, and results. This information was compared with the existing literature to assess the coherence and validity of the obtained findings [32].

This approach allowed for a thorough understanding of the current state of knowledge on the topic, identifying patterns and gaps in the literature, and establishing a solid foundation for future research.

### 2.1. Search Procedures

This narrative review followed a comprehensive bibliographic search strategy. During June, July, and August 2024, a literature search was conducted in the WoS, Dialnet, and Scopus databases using a combination of the following descriptors: caregivers and ASD, (mental health or ASD) and (ASD or family) and (ASD and caregivers) and mental health and ASD, (caregivers ASD or mental health) and (ASD or family coping strategies) and (ASD and family challenges) and (family and ASD) and (family ASD and emotions).

The initial search was restricted to full-text open-access documents categorized under “education/educational research”, specific to primary education level, and written in English or Spanish. This initial process resulted in 197 articles. After removing duplicates, the number of articles was reduced to 110 (WoS: 8; Dialnet: 87; Scopus: 15).

**Figure 1:**
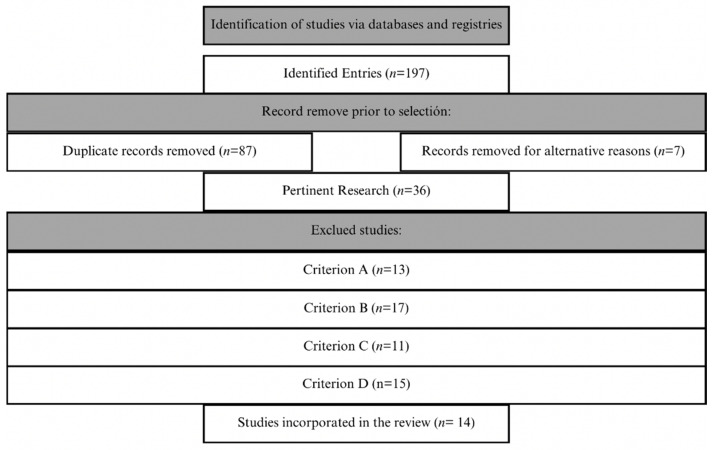
Flowchart of study selection.

In the first selection phase, the titles and abstracts of the 110 articles were reviewed, applying the inclusion criteria (A), (B), (C), and (D) described in the following section (Table 1). From this phase, 36 articles were selected for the next stage. These articles were independently evaluated by two authors to confirm their inclusion according to the defined criteria. The initial agreement between the reviewers, after the full review, was 98.81%. Discrepancies were resolved through discussion and consensus among the reviewers until achieving 100% agreement. The literature search and selection process are illustrated in Figure 1, presented using a flowchart of study selection.

Finally, 14 articles were included in this narrative review (Table 2), which were analyzed from two key perspectives: descriptive information about the studies and their findings, and the assessment of study quality and the validity of the data provided, based on inter-rater reliability. These analyses addressed several research objectives, focusing on the contribution of digital competence to the teaching–learning process of students with Autism Spectrum Disorder (ASD), as well as current educational typologies relevant to this population.

Firstly, the focus was exclusively on articles, as they are considered to offer more detailed and synthetic research on the topic. Studies in both English and Spanish were included, although the majority of the relevant articles identified were in Spanish. Additionally, the publication period was limited to between 2008 and 2024, with the aim of identifying relevant documents on the contribution of digital competence and current educational typologies in the socio-educational context of the ASD population. Finally, given the focus on the socio-educational field of individuals with ASD, the search was restricted to publications within the fields of educational, psychological, and health research.

### 2.2. Selection Criteria

To select relevant articles for the study topic, rigorous inclusion criteria were established: (A) empirical studies or research in English or Spanish; (B) free availability online; (C) focus on “education/educational research” aligned with the proposed objectives; and (D) documents centered on students with Autism Spectrum Disorder (ASD).

To ensure the scientific rigor of this review, only peer-reviewed articles were included, excluding books, book chapters, conference papers, and theses. These inclusion criteria were crucial for the main objective of the research, which is to identify studies addressing the contribution of digital competence and current educational typologies within the socio-educational context of the ASD population. Consequently, theoretical documents and those addressing educational aspects in a general manner were excluded. Additionally, studies or experiences focusing on digital competence in contexts outside the specific realm of ASD were not considered.

**Table 2:** Description of the included studies.

Author	Year	Objective	Participants	Methodology	Results
Restrepo, Castañeda, Gómez y Molina [33]	2023	Analyzed health-related quality of life, emotional distress, and caregiver burden for children with neurodevelopmental disorders during the pandemic.	132	Qualitative and Quantitative	The main findings of this study indicated alterations in the physical role, bodily pain, general health, and vitality of caregivers, in addition to moderately low levels of depression, anxiety, stress, and burden symptoms. Notable differences were identified in social functioning between the motor disability and ADHD groups, as well as in stress and burden levels between the autism and motor disability groups. Moderate correlations were also found between the dimensions of bodily pain and emotional role with levels of depression, anxiety, stress, and burden. In conclusion, while variables related to physical health showed a greater impact, the consequences related to mental health were found to be more significant and pronounced.
Durán, García, Fernández y Sanjurjo [34]	2016	Evaluated the quality of life of primary caregivers of individuals with Autism Spectrum Disorder (ASD), as well as analyzing the coping strategies employed and the level of parental stress experienced.	50	Qualitative and Quantitative	The results indicated that parental stress, coping strategies, and quality of life are not significantly associated with the developmental stage of the individual with ASD; that quality of life in the psychological, social, and environmental domains is lower in caregivers who experience clinically significant levels of parental stress; and that quality of life in the psychological domain is higher among those who employ more adaptive coping strategies, such as social support or positive reappraisal. In contrast, the use of strategies such as aggressive reaction, emotional avoidance, or inability to cope with difficulties is associated with a poorer quality of life in the psychological, social, and environmental domains.
Vega, Ocaranza y Saavedra [35]	2020	Determined the extent of the relationship between dyadic adjustment and emotional burden among caregivers of children and adolescents with Autism Spectrum Disorder (ASD).	26	Quantitative	The study revealed that 42.3% of the sample experiences some degree of emotional distress in their caregiving role. This finding supports the hypothesis that dyadic adjustment has a significant impact on the perception of emotional burden associated with caregiving for individuals with Autism Spectrum Disorder (ASD). In particular, a negative correlation was observed between dyadic adjustment and caregiver burden, suggesting that positive dyadic adjustment is associated with a lower perception of intense burden. In other words, caregivers who experience better dyadic adjustment tend to report lower levels of stress and emotional burden compared to those who face difficulties in this area. These results underscore the importance of enhancing dyadic adjustment to mitigate emotional burden in the context of caring for children with ASD, highlighting the need for interventions that promote a more harmonious relationship.
Caicedo [36]	2024	Examined the relationship between depression and guilt in primary caregivers of children with Autism Spectrum Disorder (ASD). The goal was to identify how these emotional factors interrelate and contribute to the overall experience of caregivers in order to better understand the psychological dynamics affecting them.	8	Qualitative	A relationship was identified between depression and guilt in the selected sample, with depressive symptoms tending to intensify in the presence of feelings of guilt. In particular, some participants reported that guilt significantly contributed to the development or worsening of depression. These findings suggest that guilt may be a triggering or exacerbating factor for depressive symptoms in primary caregivers of children with ASD, highlighting the need to address both depression and guilt in interventions aimed at improving their well-being.
Canseco y Vargas [37]	2020	Determined the levels of anxiety and coping styles in parents of children with Autism Spectrum Disorder (ASD), identifying the intensity of anxiety experienced by these parents and analyzing the approaches they use to manage stress and the challenges associated with caring for their children.	200	Quantitative	The state of anxiety is associated with the use of various coping styles, indicating that, in the early stages of the problem, parents tend to experience distress and avoid the situation. On the other hand, anxiety is related to all the coping styles evaluated, with problem-focused coping being the most effective style for reducing distress, as it supports a more effective management of the challenges associated with raising a child with ASD.
Bagnato, Hontou, Barbosa, Gadea [38]	2023	Expanded knowledge about parental stress experienced by primary caregivers of individuals with Autism Spectrum Disorder (ASD).	106	Qualitative and Quantitative	Inverse relationships were found between family interaction and parental role with the dysfunctional parent–child interaction factor, indicating that positive family interaction and an effective parental role are associated with lower dysfunction in the parent–child relationship. This highlights the need to integrate assessments of parental stress into interventions for individuals with Autism Spectrum Disorder (ASD), as the emotional well-being of caregivers significantly influences family quality of life.Among the limitations reported by participants, 82% experience persistent difficulties in relating due to thoughts, feelings, and behaviors; 51.9% have communication problems; and 49.1% face ongoing difficulties with learning, concentration, or memory. These barriers underscore the importance of developing specific strategies and supports to improve caregiver well-being.
Murdock, Andrade, Valdés [39]	2023	Examined the importance of emotional management in parents of children diagnosed with Autism Spectrum Disorder (ASD), and evaluated how appropriate management of emotions by these parents can positively impact their emotional well-being and the overall development of their children.	16	Qualitative	It is evident that the emotional management of parents of children with ASD does not receive adequate attention, highlighting the need to focus efforts on providing specific tools and support to help parents manage their emotions both upon receiving the diagnosis and throughout the child’s life. The research underscores the urgency of offering guidance and assistance to parents who face the particular challenges of raising a child on the autism spectrum. It is also noted that, when dealing with emotional demands and daily stresses, parents often experience a wide range of feelings, including anxiety, frustration, and stress; emphasizing this finding is the importance of integrating emotional support and management strategies for caregivers in order to improve their well-being and, consequently, the quality of care.
Seguí, Ortiz y De Diego [40]	2008	Considered the levels of caregiver burden experienced by those caring for children with Autism Spectrum Disorder (ASD), as well as their mental and physical health status.	40	Qualitative	The results reveal a high level of burden among caregivers of children with Autism Spectrum Disorder (ASD), as well as a notable deterioration in their mental and physical health compared to the general population. Positive and significant correlations were observed between levels of burden and the psychopathological and health dimensions evaluated, which is consistent with findings from previous research in this field. These results support the urgent need to develop specific care and support programs for caregivers of children with chronic conditions in order to improve their well-being and manage the associated challenges more effectively.
Hernández [41]	2022	Determined the prevalence of depression and anxiety among parents of children diagnosed with Autism Spectrum Disorder (ASD).	43	Qualitative and Quantitative	The analysis revealed that 62.80% of the participants exhibited significant symptoms of anxiety and depression. Additionally, a significant relationship was identified between these symptoms in parents and the presence of challenging behaviors in their children, particularly regarding aggression. It was observed that parents who received a brief psychoeducational intervention on managing their children’s ASD behaviors experienced a notable reduction in depression symptoms. These findings suggest that effective intervention can alleviate the negative emotional impact associated with caring for children with ASD.
Durán, García, Fernández, Martínezy García [42]	2011	Analyzed how caregivers of individuals with Autism Spectrum Disorder (ASD) perceive their parenting experience and evaluated the impact this experience has on key areas of their personal development.	50	Qualitative and Quantitative	It was found that between 70% and 74% of primary caregivers report significant changes in various aspects of their lives as a result of daily living with an individual with Autism Spectrum Disorder (ASD). These changes perceived by caregivers are closely related to high levels of parental stress, suggesting that such changes may be considered indicators of overload. In particular, feelings of despair and alterations in life expectations emerge as the dimensions most associated with parental stress in the context of the study. This indicates that, regardless of the clinical severity of perceptions, all caregivers agree on recognizing the inherent difficulties of raising a person with ASD.
Díaz [43]	2023	Analyzed the perception of family quality of life among caregivers of children with Autism Spectrum Disorder (ASD) and determined how these factors impact their ability to manage stress and maintain an optimal level of family well-being.		Quantitative	The results indicate that parental stress significantly impacts family quality of life, while social support is positively related to this quality and directly influences parental stress. It was found that coping strategies did not mediate the relationship between stress and quality of life, nor did employment status, severity of ASD, or type of family explain significant variations in family quality of life. These findings underscore the importance of maintaining a high quality of life despite the challenges associated with ASD and highlight the need to strengthen support systems to provide greater security and well-being.
Ezcurra [44]	2022	Evaluated the levels of depression and quality of life in mothers of children with Autism Spectrum Disorder (ASD), and described the sociodemographic characteristics of these mothers to gain a comprehensive understanding of their situation and condition.	36	Qualitative and Quantitative	The study reveals that 64.29% of homemakers experience persistent feelings of sadness, and 74% express a sense of disappointment with themselves, indicating a high level of severe depression (86.5%). The quality of life is notably poor, with 45% rating their health as fair and 30% as poor. Additionally, 70% experience significant limitations in physical exertion, 59% have ceased engaging in activities due to health problems, and 58.5% have stopped activities for emotional reasons. These findings underscore the substantial impact of depression and health issues on the daily lives of mothers of children with Autism Spectrum Disorder (ASD).
Zapata [45]	2021	Investigated the relationship between caregiver burden and stress coping strategies in parents of children with Autism Spectrum Disorder (ASD).	35	Qualitative and Quantitative	According to the research findings, although caregivers did not exhibit high levels of overall burden, the observed levels of burden are associated with the use of coping strategies that are not fully effective. In other words, while the extent of the burden is not extremely high, the strategies employed by caregivers to manage this burden are not always the most suitable. This results in the inadequate handling of situations, which may lead to a lack of training and information that might not provide the necessary relief.
Astocóndor [46]	2023	Analyzed coping strategies for stress and the extent of emotional burden in parents of children with Autism Spectrum Disorder (ASD).	100	Quantitative	Although high levels of caregiver burden were not generally found, the observed levels of burden are associated with the use of maladaptive coping strategies in caregivers of individuals with Autism Spectrum Disorder (ASD). The findings indicate a significant relationship between burden and coping strategies related to planning, self-control, responsibility, and avoidance. Additionally, it was found that beliefs about caregiving significantly influence the use of coping strategies such as self-control, responsibility, and avoidance, suggesting that personal perceptions and beliefs have a notable impact on how caregivers manage emotional burden.

## 3. Results

### Identification of the Selected Publications

In the category of Quality of Life, it was observed that, although caregivers report moderate levels of depression and anxiety, they also experience high levels of physical distress. This suggests that the overall well-being of caregivers is compromised in ways that may not always be evident in mental health evaluations. Durán et al. [34] note that caregivers facing significant stress have a lower quality of life in psychological and social domains, which can lead to an inability to engage in recreational and social activities that are fundamental for their emotional well-being. Furthermore, Ezcurra [44] highlights that a high percentage of mothers feel constantly sad, indicating a considerable prevalence of depression that impacts not only their daily lives but also their capacity to care for and support their children.

Regarding Stress and Emotional Burden, it was observed that the quality of family interaction correlates negatively with parental stress. According to Bagnato et al. [38], caregivers who enjoy positive family relationships tend to experience lower levels of stress, suggesting that family dynamics can act as an emotional buffer. However, Hernández [41] finds that a significant percentage of parents exhibit symptoms of anxiety and depression, particularly those facing challenging behaviors in their children. Murdock et al. [39] reinforce this idea by stating that a lack of adequate emotional support negatively affects the well-being of caregivers and, consequently, the development of their children. This finding underscores the interconnectedness between the caregiver’s well-being and the family environment in which the child is raised.

The category of Coping Strategies emphasizes the importance of the styles that caregivers employ to manage stress. Canseco and Vargas [37] identify that problem-focused coping is the most effective for mitigating anxiety, suggesting that caregivers who adopt proactive approaches, such as seeking social support and re-evaluating their situations, tend to experience less emotional distress. In contrast, Zapata [45] and Astocóndor [46] warn that the use of ineffective strategies, such as avoidance and excessive self-control, can lead to a greater emotional burden, indicating the need for interventions that equip caregivers with adaptive coping methods.

In the section on Family Impact, it is documented that positive dyadic adjustment is associated with lower levels of emotional burden in caregivers. Vega et al. [35] demonstrate that healthy family relationships are a crucial factor for the well-being of caregivers, suggesting that promoting harmonious family interactions should be a priority in interventions. Additionally, Seguí et al. [40] indicate a significant deterioration in the mental and physical health of caregivers compared to the general population, reinforcing the need for a comprehensive approach that addresses both their emotional and physical needs.

Finally, the category of Emotions and Well-being highlights that emotional factors such as guilt exacerbate the distress experienced by caregivers. Caicedo [36] indicates that guilt among caregivers correlates with an increase in depressive symptoms, acting as a significant trigger for emotional distress. Durán et al. [42] identify that significant changes in caregivers’ lives, associated with high levels of stress, suggest that interventions are needed to comprehensively address both depression and guilt. This approach is crucial for improving caregivers’ quality of life and, consequently, the care they can provide to their children with ASD.

Table 3, presented below, summarizes the key findings derived from the narrative review on the well-being of caregivers of children with Autism Spectrum Disorder (ASD). This table organizes the main categories (Quality of Life, Stress and Emotional Burden, Coping Strategies, Family Impact and Emotional Well-being), accompanied by the relevant studies that support each category. Its structure allows for a clear and concise observation of the interrelated factors that affect both the emotional health and quality of life of caregivers, facilitating a comprehensive understanding of the impact and specific support needs for this population.

## 4. Discussion

The narrative review of studies on the well-being of caregivers of children with Autism Spectrum Disorder (ASD) reveals a complex and multifaceted picture of the challenges these individuals face. The reviewed studies address various aspects, including quality of life, emotional distress, coping strategies, and the impact of dyadic adjustment and family relationships. This discussion aims to integrate and compare the findings to provide a comprehensive view of how these factors influence the caregivers’ experience.

The results show notable variability in the quality of life among caregivers. Evidence suggests that caregivers of children with ASD face a range of interrelated challenges that affect their overall well-being, with a combination of significant alterations in both physical and emotional health. Studies have documented that, despite some caregivers reporting moderately low levels of depression, anxiety, and stress, the impact on their mental health may be deeper than these levels suggest. This discrepancy might reflect both adaptive mechanisms developed by the caregivers and an underestimation of the actual emotional impact.

In the study conducted by Restrepo et al. [33], significant alterations in variables related to caregivers’ physical and emotional health were identified during the pandemic, although reported levels of depression, anxiety, and stress were moderately low. These findings suggest that, despite the high physical alterations, the impact on mental health may be deeper and more pronounced than caregivers are willing to report. This aligns with the stress and adaptation theory [47], which posits that prolonged exposure to stressors can deteriorate both physical and psychological well-being. The discrepancy between physical distress and reported emotional distress may indicate that caregivers are developing adaptive mechanisms or that there is an underestimation of the actual emotional impact.

Similarly, studies by Seguí et al. [40] and Hernández [41] corroborate the negative impact on mental and physical health, reporting high levels of overload and significant deterioration compared to the general population. They also show that a considerable percentage of caregivers exhibit significant symptoms of anxiety and depression. The relationship between the presence of challenging behaviors in children and depressive symptoms further underscores the importance of addressing these emotional aspects in interventions. The findings suggest that emotional distress and health problems may be interrelated, highlighting the need for comprehensive care that considers both physical and emotional aspects of caregiver well-being.

Regarding coping strategies, they play a crucial role in managing stress and overload. Studies show that adaptive strategies, including social support and positive reappraisal, are linked to improved quality of life and lower emotional overload. In contrast, the use of maladaptive strategies, such as avoidance and self-control, is linked to greater overload and emotional distress. This evidence highlights the importance of promoting effective and adaptive coping strategies to help caregivers better manage the stress associated with caring for a child with ASD.

In this regard, Durán et al. [34,42] identified that adaptive coping strategies, such as social support and positive reappraisal, are associated with a better quality of life. This is consistent with the adaptive coping theory [48], which suggests that strategies that facilitate positive adaptation and problem reappraisal are more effective in reducing stress. On the other hand, Zapata [45] and Astocóndor [46] found that the use of suboptimal and maladaptive coping strategies, such as avoidance and self-control, is associated with increased emotional overload. These maladaptive strategies may exacerbate emotional burden, underscoring the need for interventions that promote the use of adaptive strategies to improve stress management.

A crucial aspect that should not be overlooked is the impact of the specific variables of autistic symptomatology on the emotional state of caregivers. The severity of the symptoms and the presence of problematic behaviors and issues related to the child’s autonomy are factors that can significantly influence the overall well-being of the family. Studies such as that of Iovino et al. [49], who explore the management of problem behaviors by adapting the IISCA, highlight the importance of addressing these variables in interventions. Ornstein and Gaugler [50] have documented how problematic behaviors are associated with higher levels of emotional overload in caregivers of people with dementia, a finding that could be extrapolated to caregivers of children with ASD. It is essential to consider these variables when comparing the emotional burden between different conditions and develop more personalized interventions that improve the well-being of families.

The study by Canseco and Vargas [37] also emphasizes the importance of coping styles, showing that problem-focused coping is the most effective in mitigating distress. This is also consistent with the aforementioned coping theory, which distinguishes between problem-focused and emotion-focused strategies. The results suggest that promoting proactive and solution-oriented approaches can be beneficial for caregivers.

Dyadic adjustment and family relationships also have a significant impact on caregivers’ well-being. Studies have shown that positive dyadic adjustment and healthy family interactions are associated with lower levels of overload and emotional stress. Emotional and practical support within the family environment acts as a buffer against stress, enhancing caregivers’ quality of life. Therefore, strengthening family relationships and providing adequate support from diagnosis through the parenting process are essential for improving caregivers’ overall well-being.

Studies by Vega et al. [35] and Bagnato et al. [38] show that positive dyadic adjustment and effective family interaction are associated with lower levels of overload and emotional stress. Dyadic adjustment theory [51] and social support theory [52] explain how positive interpersonal relationships and emotional support can reduce emotional burden. The importance of family support is reflected in the studies by Murdock et al. [39] and Díaz [43], which highlight that good social and emotional support can alleviate parental stress and improve quality of life. This suggests that interventions aimed at improving communication and family support are crucial for reducing overload and enhancing overall well-being [53].

Lastly, studies also emphasize the need for specific interventions that address both depression and guilt, as observed in Caicedo’s study [36], which reveals that guilt can exacerbate depressive symptoms. The lack of adequate support to manage emotions, as noted in Murdock et al.‘s work [39], underscores the importance of providing specific tools from diagnosis through the parenting process.

In summary, the narrative review underscores the need for a comprehensive approach to address the well-being of caregivers of children with ASD. Combining adaptive coping strategies, strengthening dyadic adjustment, and enhancing family support are crucial for improving quality of life and reducing emotional overload. Integrating these approaches can provide a solid foundation for developing more specific and effective support strategies, paving the way for better stress management and greater well-being for caregivers and their families.

## 5. Conclusions

In conclusion, recent research highlights the complex challenges faced by caregivers of individuals with Autism Spectrum Disorder (ASD) and their families. The quality of life for these caregivers is significantly impacted by various interconnected factors that extend beyond immediate physical and emotional issues. While some studies report moderate levels of depression, anxiety, and stress among caregivers, this does not fully reflect the emotional burden they experience. This discrepancy may be due to the adaptive mechanisms that caregivers employ to manage their distress or an underestimation of their emotional struggles.

Coping strategies play a crucial role in managing stress and emotional overload. Evidence suggests that adaptive strategies, such as seeking social support and positive reappraisal, correlate with a better quality of life, whereas maladaptive strategies, such as avoidance, may worsen emotional distress. These findings underscore the necessity of proactive solution-oriented approaches and highlight the importance of providing sufficient resources and training for caregivers.

Family dynamics, particularly dyadic adjustment, are also essential for caregivers’ well-being. Positive family interactions are linked to lower stress levels, highlighting the need to strengthen family relationships and provide ongoing emotional support. Comprehensive efforts are required to enhance caregivers’ well-being, recognizing the complexity of their challenges and addressing them holistically.

Future research should deepen our understanding of stress and adaptation mechanisms among caregivers while implementing effective support programs. By improving support systems for caregivers, we can enhance their quality of life and foster a more understanding environment for families affected by ASD.

Finally, concerning the initial questions posed and the research objectives outlined, the following conclusions can be drawn:

- How does Autism Spectrum Disorder (ASD) affect the overall quality of life of primary and secondary caregivers compared to caregivers of children with other neurodevelopmental disorders?

The impact of Autism Spectrum Disorder (ASD) on caregivers’ quality of life is profound and multifaceted, with significant implications for both physical and emotional well-being. Caregivers of children with ASD often face a considerable burden due to the chronic and varied nature of the disorder’s symptoms, which can include communication difficulties, behavioral issues, and social skill deficits. These challenges necessitate intensive levels of attention and support, which can negatively affect caregivers’ overall quality of life.

Compared to caregivers of children with other neurodevelopmental disorders, such as Attention-Deficit/Hyperactivity Disorder (ADHD) or motor disabilities, those caring for children with ASD frequently report greater emotional overload and reduced quality of life. This is attributed to a combination of factors including communication difficulties, repetitive behaviors, and behavioral crises, which can amplify stress and fatigue. While neurodevelopmental disorders can present their own challenges, the symptoms of ASD often require more complex interventions and constant attention, leading to greater disruption in caregivers’ daily lives.

Current studies have shown that caregivers of children with ASD tend to experience a significantly affected overall quality of life, with elevated levels of stress and a higher incidence of mental health issues compared to those caring for children with other neurodevelopmental disorders. This underscores the need for more specific and tailored support approaches for these caregivers, addressing both the caregiving demands and the emotional and physical impact associated with ASD.

- What effects does caring for a child with Autism Spectrum Disorder (ASD) have on family dynamics and interpersonal relationships?

Caring for a child with Autism Spectrum Disorder (ASD) significantly impacts family dynamics and interpersonal relationships within the home. This impact can manifest in various ways, affecting the quality of relationships among family members and altering the family structure.

One of the most notable effects is the increase in stress and tension between parents, which often translates into conflicts and a decrease in marital satisfaction. The emotional burden and responsibilities associated with caring for a child with ASD can lead to greater dissatisfaction and disagreements between parents on how to handle care and necessary interventions. This stress can also affect relationships with other family members, such as siblings of the child with ASD, who may experience feelings of neglect or resentment due to the focused attention on their sibling with ASD.

Additionally, social interactions outside the immediate family may be affected, as caregivers may struggle to maintain social relationships due to the time and energy demands of caregiving. Limited time for social activities and the stigma associated with the child’s behaviors in public settings can contribute to a heightened sense of isolation and a diminished social support network.

Overall, caring for a child with ASD can lead to a reconfiguration of family dynamics and an impact on interpersonal relationships, highlighting the importance of providing support at both the family and social levels to mitigate these effects and strengthen family cohesion.

- What factors contribute to variability in the quality of life of caregivers, and how do different coping strategies affect the family members or caregivers of individuals with Autism Spectrum Disorder (ASD)?

Variability in the quality of life among caregivers of children with Autism Spectrum Disorder (ASD) can be influenced by multiple factors, including the type and severity of ASD, the level of available social support, the coping strategies employed, and the personal characteristics of the caregiver.

A key factor is the severity of the ASD symptoms. Caregivers of children with more severe or complex symptoms tend to report higher emotional burden and lower quality of life. The frequency and intensity of the child’s challenging behaviors, as well as the need for constant support, can significantly increase caregivers’ stress and fatigue.

Coping strategies play a crucial role in how caregivers manage the stress associated with caring for a child with ASD. Adaptive strategies, such as utilizing social support networks, seeking professional help, and positive reappraisal of the situation, are associated with a better quality of life and more effective stress management. Conversely, maladaptive strategies, such as avoidance or excessive self-control, can exacerbate emotional overload and contribute to a reduced quality of life.

Additionally, personal characteristics of the caregiver, such as resilience, coping skills, and access to resources, also affect variability in quality of life. Caregivers with strong support networks, adequate financial resources, and effective stress management skills tend to experience a relatively better quality of life.

These findings underscore the importance of providing personalized interventions that not only address the needs of the child with ASD, but also strengthen coping skills and the support available to caregivers. To achieve this, it is crucial that these interventions take into account the individual characteristics of parents or caregivers. These principles are reflected in the narrative review by Simeoli et al. [53], which highlights how adapting ABA-based treatments according to the needs and characteristics of caregivers can be key to improving quality of life and reducing the emotional burden associated with caring for a child with ASD. Identifying and reinforcing effective coping strategies tailored to these factors can significantly impact caregiver well-being [49].

## Figures and Tables

**Table 1:** Selection criteria.

	Inclusion Criteria	Exclusion Criteria
A	Empirical studies or research published in peer-reviewed scientific journals in English or Spanish	Other publications (documents with theoretical content or related to non-educational aspects, books, etc.)
B	Open access through the Internet	No open access through the Internet
C	Categories restricted to “education/educational research” related to the proposed objectives	Documents related to other aspects not included in the objectives of this study
D	Documents specific to family members and individuals with Autism Spectrum Disorder (ASD)	Studies or experiences involving other populations

**Table 3:** Maps of evidences.

Category	Reference	Year	Method	Key Findings
Quality of Life	Restrepo et al. [33]	2023	Mixed	Identification of high levels of physical distress, with depression and anxiety reported at moderate levels.
	Durán et al. [34]	2016	Mixed	Caregivers with significant stress report a lower quality of life in psychological and social areas.
	Ezcurra [44]	2022	Mixed	A high percentage of mothers report symptoms of sadness and elevated levels of depression, impacting their quality of life.
Stress and Emotional Burden	Bagnato et al. [38]	2023	Mixed	The quality of family interaction is negatively correlated with parental stress.
	Hernández [41]	2022	Mixed	A high percentage of parents exhibit significant symptoms of anxiety and depression, related to challenging behaviors.
	Murdock et al. [39]	2023	Qualitative	Parents lack adequate emotional support, which affects their well-being and the development of their children.
Coping Strategies	Canseco and Vargas [37]	2020	Quantitative	Coping styles impact anxiety levels; problem-focused approaches are found to be more effective.
	Zapata [45]	2021	Mixed	Although the burden is not critical, ineffective coping strategies are prevalent.
	Astocóndor [46]	2023	Quantitative	Maladaptive strategies are associated with high levels of emotional burden; personal beliefs influence coping.
Family Impact	Vega et al. [35]	2020	Quantitative	Positive dyadic adjustment is associated with lower emotional burden in caregivers; there is a need to strengthen relationships.
	Díaz [43]	2023		Maladaptive strategies are associated with greater emotional burden in caregivers, while personal beliefs influence their coping methods.
	Seguí et al. [40]	2018	Qualitative	Significant deterioration in the mental and physical health of caregivers is observed compared to the general population.
Emotions and Well-Being	Caicedo [36]	2024	Qualitative	Guilt among caregivers correlates with an increase in depressive symptoms, acting as a significant trigger.
	Durán et al. [42]	2011	Mixed	Caregivers’ lives are affected by high levels of stress and significant changes in their overall well-being.
